# Device updates successfully reduce T‑wave oversensing and inappropriate shocks in subcutaneous ICD patients

**DOI:** 10.1007/s12471-018-1160-y

**Published:** 2018-09-24

**Authors:** R. Larbig, L. J. Motloch, M. Bettin, A. Fischer, N. Bode, G. Frommeyer, A. Loeher, J. Koebe, F. Reinke, L. Eckardt

**Affiliations:** 1 Division of Electrophysiology, Department of Cardiovascular Medicine, University of Muenster, Muenster, Germany; 2 Department of Cardiac and Thoracic Surgery, University of Muenster, Muenster, Germany; 3 Department of Internal Medicine II, Paracelsus Medical University, Salzburg, Austria

**Keywords:** S-ICD, Oversensing, Inappropriate shocks

## Abstract

**Aims:**

To analyse the impact of device and software updates on the prevention of T‑wave oversensing (TWOS) and inappropriate shocks (IS) in subcutaneous ICD (S-ICD) patients.

**Background:**

TWOS is a feared complication after implantation. It may lead to harmful IS. To date, specific strategies to reduce these events are lacking.

**Methods:**

In this retrospective single-centre trial we analysed 146 S‑ICD patients who were implanted between 2010 and 2016. In all eligible consecutive patients (*n* = 139), follow-up of at least 6 weeks was studied. The incidence of TWOS/IS was analysed in patients receiving a 2^nd^ generation S‑ICD (Emblem-S-ICD) between 2014 and 2016 (Emblem). Their outcome was compared with a control group (SQ) treated with the SQ1010 device between 2010 and 2014, who were followed up for a maximum of 2 years. Furthermore, to test if the software update SMR8 reduces inappropriate shocks in the SQ1010-S-ICD population, the incidence of TWOS/IS was evaluated before and after update installation.

**Results:**

Basic characteristics and indications for S‑ICD implantation were similar in both groups. However, the cumulative incidence of TWOS/IS was significantly decreased in Emblem vs. SQ (SQ: 15.4%, *n* = 14/91 vs. Emblem 4.2%, *n* = 2/48; *p* = 0.049). Furthermore, with regards to the SQ population we also observed a trend towards a significant reduction of TWOS/IS after installation of the software update SMR8 in 2014 (before update: 13.4%, *n* = 11/82 vs. after update: 4.6%, 3/65, *p* = 0.07).

**Conclusion:**

2^nd^ generation devices but probably also the SMR8 software update reduce the incidence of TWOS/IS in S‑ICD patients.

## What’s new?

- Updated S‑ICD devices successfully reduce the incidence of TWOS/IS in S‑ICD patients.
- Probably the SMR8 software update also successfully reduces the incidence of TWOS/IS in S‑ICD patients.
- These findings stress the need for constant improvement of the S‑ICD software and underline the therapeutic value of detection algorithms in S‑ICD patients.

## Introduction

The implantable cardioverter-defibrillator (ICD) has been an important treatment option for selected patients who are at risk of sudden cardiac death [1]. However, despite the recognised mortality benefit, perioperative complications such as pneumothorax, cardiac perforation, and tamponade as well as long-term technical difficulties such as lead failure or device infection have become important issues in clinical practice [2–6].

In 2009 the totally subcutaneous implantable defibrillator (S-ICD) was introduced as a new therapeutic alternative for suitable patients (the S‑ICD is not suitable for patients with a pacing/CRT indication and less preferable for patients with (monomorphic) VTs who could otherwise be treated with anti-tachycardia pacing). The early results from the EFFORTLESS registry demonstrated appropriate system performance with clinical event rates comparable with those reported for conventional transvenous ICD systems [7]. Although implantation of the S‑ICD reduces implant-related complications, a high rate of inappropriate shocks (IS) with tremendous impact on quality of life was reported [8, 9]. These potentially harmful events are mostly triggered by T‑wave oversensing (TWOS) which is a feared complication after S‑ICD implantation [9–11]. Hypertrophic cardiomyopathy has been linked to TWOS [12, 13]. Nevertheless, so far, little is known on how to predict or prevent inappropriate shocks despite specific ECG parameters [12]. Recently, a new double-detection algorithm was introduced to reduce the incidence of T‑wave oversensing [13]. The new double-detection algorithm was available as software update SMR8 for the first generation SQ-S-ICD devices. The new detection algorithm analyses the morphology of three consecutive QRS complexes in order to specifically minimise TWOS and is usually automatically uploaded during follow-up visits. Besides, in 2015 the new S‑ICD generation with this double-detection algorithm as standard, the Emblem-S-ICD, became available. Whether these new strategies have an impact on the incidence of TWOS/IS remains unclear.

Therefore, we hypothesised that the new generation device, the Emblem-S-ICD, as well as the software update SMR8, might reduce the incidence of these harmful events.

## Methods

### Study cohort

In this retrospective single-centre trial we screened the data of 146 patients who were treated with S‑ICD implantation between January 2010 and October 2016. Patients either received the first generation SQ1010-S-ICD or the new Emblem-S-ICD. Patients were only included in the analyses if at least a complete follow-up (FU) of 6 weeks was present. In the eligible study cohort of 139 patients, medication, results from diagnostic tests and history of concomitant diseases were obtained from the university patient database. Importantly, records and S‑ICD interrogation results of all FU visits were evaluated and patients were screened for the incidence of TWOS and IS. Since only one patient in our cohort (implanted with a SQ1010 device) experienced IS in the absence of TWOS, we also considered isolated episodes of TWOS without IS to be clinically relevant, especially since the S‑ICD provides no manual adjustability of the S‑ICD ECG sensitivity. Therefore, we chose a combined endpoint of these events (TWOS/IS). Specifically, TWOS/IS was defined as at least one episode of TWOS and/or IS during the period of FU. To analyse the impact of device type on the incidence of TWOS/IS, FU up to 2 years (mean FU: 500 ± 267 days) after implantation was studied. Outcome of patients receiving the new Emblem-S-ICD (Emblem; *n* = 48) was compared with the performance of the first-generation device SQ1010-S-ICD (SQ; *n* = 91). Furthermore, to test if the new double-detection algorithm SMR8 (available since August 2014 in our cohort) reduces TWOS/IS in the SQ1010-S-ICD population, patient outcome after implantation (SQ-SMR8(−); *n* = 82) and/or after update installation (SQ-SMR8(+); *n* = 65) was evaluated for up to 2 years (mean FU: 551 ± 168 days). Patients were only included in the analysis if a least a complete FU of 6 weeks after implantation and/or after software update installation could be obtained. With respect to patient safety and privacy all identities in this retrospective study were excluded from the manuscript.

### Selection of patients for S‑ICD and S‑ICD implantation

Upon admission, all patients with either a primary or secondary prophylactic S‑ICD indication underwent a thorough physical examination by a physician. Furthermore, information about the clinical history, medication and concomitant diseases was obtained. Laboratory data were gathered and a 12-lead ECG was recorded. Additionally, patients were screened for appropriate ECG morphology according to the manufacturers’ guidelines in order to be eligible for subsequent S‑ICD implantation. Finally, a chest X‑ray was performed. The S‑ICDs were inserted as previously described [14].

### Device follow-up

Device performance was evaluated after implantation before discharge from our hospital. Second evaluation was initiated 6 weeks after implantation and then in periods of 6 months or earlier if the patient experienced shocks or any complications. An experienced electrophysiologist performed device FU and programming. As soon as available (August 2014) SMR8 software was updated on regular FU visits in all SQ patients.

### Statistical analysis

Statistical analyses were performed using SPSS 22 software (IBM, Armonk, USA). The results are given as mean ± standard deviation (SD). Differences between groups and subgroups were evaluated by chi-square testing for discrete variables and Student’s t test for continuous variables. For ordinal data the Mann-Whitney U test was used. A *p* < 0.05 was considered statistically significant.

## Results

The basic characteristics of our study cohort are presented in Table 1.

**Table 1:** Baseline characteristics

	SQ1010®-SICD (*n* = 91)		Emblem®-SICD (*n* = 48)		P-values
	*n*	% or Mean ± SD	*N*	% or Mean ± SD	
Male gender	59	64.8%	35	72.9%	*p* = 0.333
Age (years)	91	41.4 ± 15.3	48	44.1 ± 15.7	*p* = 0.325
*Cardiac pathology*					*p* = 0.291
Coronary artery disease	12	13.2%	9	18.8%	
Dilated cardiomyopathy	15	16.5%	5	10.4%	
Electrical heart disease	17	18.7%	10	20.8%	
Hypertrophic cardiomyopathy	21	23.1%	5	10.4%	
Congenital heart disease	6	6.6%	6	12.5%	
Valvular heart disease	7	7.7%	2	4.2%	
Idiopathic ventricular fibrillation	7	7.7%	4	8.3%	
Cardiac sarcoidosis	1	1.1%	0	0.0%	
Other	5	5.5%	7	14.6%	
*Primary prevention*	45	49.5%	21	43.8%	*p* = 0.522
*Secondary prevention*	46	50.5%	27	56.3%	
LVEF (%)	62	51.6 ± 14.7	61	51.6 ± 13.2	*p* = 0.990

Comparisons between groups were performed with Chi-squared test for nominal, and Student’s t‑test for rational variables

*LVEF* left ventricular ejection fraction by biplane Simpson’s method using transthoracic echocardiography

*P* < 0.05 was regarded statistically significant

**Fig. 1:**
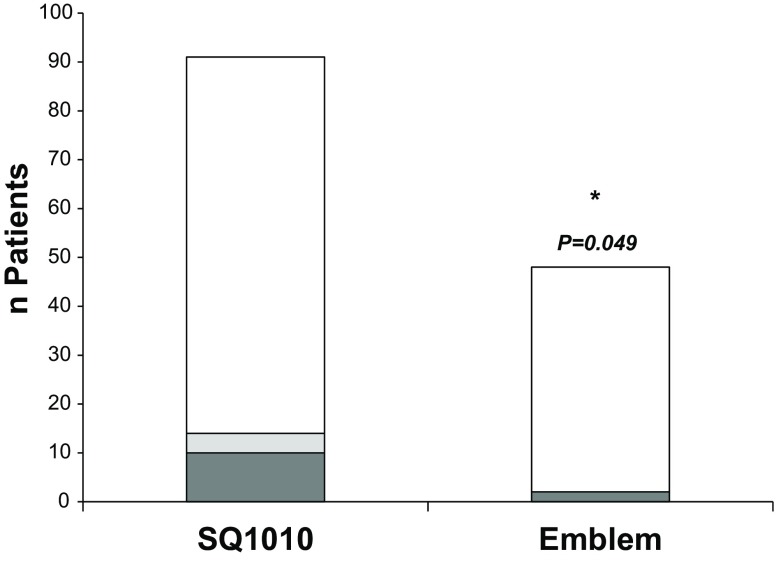
TWOS/IS incidence in Emblem vs. SQ S‑ICDs. Incidence of TWOS/IS was significantly reduced in the Emblem-S-ICD (*n* = 48) vs. SQ1010-S-ICD (*n* = 91) population. FU up to 2 years was evaluated (*White* = no TWOS/IS; *grey* = TWOS/IS (*dark grey* = TWOS leading to IS or IS independent of TWOS: *light grey* = TWOS only), *P < 0.05 using χ^2^ test)

**Fig. 2:**
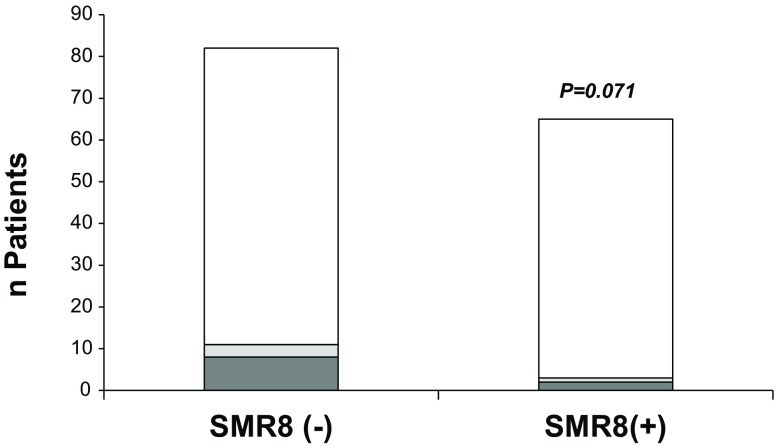
TWOS/IS incidence in SQ ± SMR8 update. Analysis of the incidence of TWOS/IS after SQ1010-S-ICD implantation (SQ-SMR8 (−); *n* = 82) vs. outcome after SQ1010-S-ICD software SMR8 update installation (SQ-SMR8(+); *n* = 65). We observed a strong trend towards a significant reduction in the incidence of TWOS/IS in SQ-SMR8(+). A FU up to 2 years after implantation and/or after update installation was evaluated (*White* = no TWOS/IS; *grey* = TWOS/IS (*dark grey* = TWOS leading to IS or IS independent of TWOS: *light grey* = TWOS only), *P < 0.05 using chi-square-test)

**Fig. 3:**
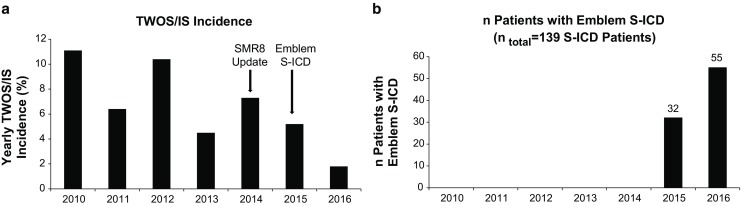
One-year incidence of TWOS/IS and proportion of patients implanted with Emblem-S-ICD.** a** Analysis of the 1‑year incidence of TWOS/IS between 2010 and 2016. **b** One-year proportion of patients implanted with Emblem-S-ICD in our study population. A trend towards reduction of TWOS/IS within implementations of new software (SMR8 update August 2014) and device update was observed. The lowest 1‑year incidence of TWOS/IS was revealed in 2016 when the proportion of Emblem-S-ICD implanted patients was highest (55 patients) and all SQ1010-S-ICD patients were already updated with the SMR8 software

No significant differences between gender, age and cardiac function indicated by left ventricular ejection fraction were observed. Furthermore, the reasons for S‑ICD treatment were similar in both groups. However, major differences in the incidence of TWOS/IS were uncovered since the Emblem patients presented a significantly lower incidence of TWOS/IS (SQ: 15.4%, *n* = 14/91 vs. Emblem 4.2%, *n* = 2/48; *p* = 0.049, Fig. 1). In addition, also in the SQ population a strong trend towards a reduction of TWOS/IS was observed when devices were updated with the new SMR8 software (SQ-SMR8(−): 13.4%, *n* = 11/82 vs. SQ-SMR8(+): 4.6%, 3/65, *p* = 0.07; Fig. 2). (4) Based on our findings we concluded that, indeed, the change in device generation but also probably software updates successfully reduced the incidence of TWOS/IS in S‑ICD patients. To further investigate this issue, we evaluated the 1‑year incidences of TWOS/IS in our study cohort. Indeed, between 2010 and 2016, we were able to uncover a reduction of these harmful events after implementation of the new software (SMR8 update August 2014) and device updates (Emblem-S-ICD implantation starting January 2015; Fig. 3; mean 1‑year incidence of TWOS/IS 2010–2014 = 7.94 ± 2.77% vs. mean 1‑year incidence of TWOS/IS 2015–2016 = 3.50 ± 2.40%). Notably, the lowest 1‑year incidence of TWOS/IS of 1.8% was observed in 2016 when 55 out of 139 S‑ICD patients had an Emblem-S-ICD and all SQ1010-S-ICD patients were already updated with the SMR8 software (Fig. 3).

## Discussion

TWOS and IS are severe complications after S‑ICD implantation [9]. They are known to severely traumatise patients [8]. Notably, these harmful events are mostly triggered by TWOS [7, 10, 11]. Hence, development of effective strategies to prevent TWOS/IS is highly desirable. In recent years new generation S‑ICD devices with potentially better T‑wave signal sensing characteristics were introduced. Therefore, we sought to analyse the performance of the new Emblem-S-ICD in comparison with the first generation SQ1010-S-ICD. Indeed, our results obtained during a real-life scenario were able to detect a significant reduction of TWOS/IS in patients implanted with the Emblem-S-ICD. Notably, TWOS promotes IS, and therefore it is known to reduce the quality of life.

Another important finding of our trial is a trend towards a significant reduction of TWOS/IS in SQ1010-S ICD patients who, during FU, received the signal optimising software update SMR8. This update implements a new double-detection algorithm which analyses the morphology of three consecutive QRS complexes in order to specifically minimise TWOS and is usually automatically uploaded during follow-up visits. Of note, our observation was also consistent with reports on the impact of software updates on the incidence of IS for transvenous ICDs (15). However, our results did not reach statistical significance. This could probably be attributable to a too low number of patients in this group. Our suggestion is also underlined by our more detailed analysis of the yearly TWOS/IS incidence. In our collective we were able to show the correlation between a reduced 1‑year incidence and the combination of device updates and an increase in the absolute number of the new Emblem-S-ICD in all our S‑ICD patients. Notably, a 1-year event rate of 1.8% was observed in 2016, when the absolute number of Emblem-S-ICD implanted patients was highest and all SQ1010-S-ICDs were updated with the SMR8 software. Of note, the observed incidence is comparable to event rates reported for the latest transvenous ICD models [15]. This is in accordance with latest S‑ICD trials, which also showed a comparable rate of inappropriate shocks in S‑ICD and ICD study cohorts (Table 2; [9]) Therefore, the S‑ICD seems be at least equal in performance while not having lead-associated complications in comparison with a transvenously implanted ICD. Also, our results stress the importance of software algorithms as an effective medical tool for treatment of TWOS/IS. Our findings hint at the great potential of the S‑ICD for suitable patients since therapeutic efficiency was sufficient in previous trials [7]. However, our suggestions should be confirmed by larger randomised prospective multicentre trials.

**Table 2:** Incidence of T‑wave oversensing and inappropriate shocks

	SQ1010®-SICD (*n* = 91)		Emblem®-SICD (*n* = 48)		P-values
	*n*	%	*N*	%	
*Total*	14/91	15.4%	2/48	4.2%	*p* = 0.049
*Cardiac pathology*					
Coronary artery disease	1/12	8.3%	0/9	0.0%	*p* = 0.375
Dilated cardiomyopathy	1/15	6.7%	1/5	20.0%	*p* = 0.389
Electrical heart disease	3/17	17.6%	0/10	0.0%	*p* = 0.159
Hypertrophic cardiomyopathy	8/21	38.1%	0/5	0.0%	*p* = 0.097
Congenital heart disease	0/6	0.0%	0/6	0.0%	n. a.
Valvular heart disease	0/7	0.0%	0/2	0.0%	n. a.
Idiopathic ventricular fibrillation	0/7	0.0%	1/4	25.0%	*p* = 0.165
Cardiac sarcoidosis	0/1	0.0%	0/0	0.0%	n. a.
Other	1/5	20.0%	0/7	0.0%	*p* = 0.217

Comparisons between groups were performed with Chi-squared test

*n. a.* not available

*P* < 0.05 was regarded as statistically significant

### Limitations

Our study suffers from several limitations. Generally, results obtained retrospectively in a single centre should be confirmed in a preferably multicentre, randomised prospective study. However, our data represent a real-life scenario since they were obtained during daily clinical practice. Incidence of TWOS/IS in early SQ1010-S-ICD patients could be overestimated since clinical experience over time might have improved patient care, especially with regards to the selection of patients for S‑ICD implantation. Also, as indicated by the analysis of the patients’ baseline parameters, underlying pathologies did not significantly differ between groups; this may be related to the small number of patients in our study cohort. HCM seems slightly more prevalent in the SQ1010 group (23.1 vs. 10.4%, *p* > 0.05) without reaching statistical significance. This issue may affect the outcome parameter of TWOS/IS between the two groups. Although we tried to present similar FU periods in all groups, one has to keep in mind that FU in some patients implanted with the Emblem-S-ICD in late 2016 was shorter as compared with the SQ1010-S-ICD, which might lead to underestimation of the TWOS/IS in this population.

## Conclusion

In summary our results indicate that an updated device and probably a software update successfully reduce the incidence of TWOS/IS in S‑ICD patients suggesting S‑ICD as a safe therapeutic option for primary and secondary prevention of SCD in suitable patients.
